# A case report of bladder and intestinal endometriosis, and the relationship between sex hormone receptor expression and PIK3CA mutation analysis

**DOI:** 10.1186/s12905-021-01269-6

**Published:** 2021-03-21

**Authors:** Akiko Kanto, Yasushi Kotani, Kosuke Murakami, Chiho Miyagawa, Hidekatsu Nakai, Noriomi Matsumura

**Affiliations:** Department of Obstetrics and Gynecology, Kindai University Faculty of Medicine, 377-2 Ohno-higashi, Osaka-sayama, Osaka 589-8511 Japan

**Keywords:** Endometriosis, PIK3CA mutation, Progesterone resistance

## Abstract

**Background:**

Extragonadal endometriosis is a rare condition, and its disease manifestation and long-term prognosis have not been elucidated. We report an extragonadal endometriosis case controlled by drug therapy for 14 years with analysis of the sex hormone receptor expression and PIK3CA mutation.

**Case presentation:**

The patient was diagnosed with bladder endometriosis at age of 30 years, and underwent bilateral nephrostomy and GnRHa therapy with add-back therapy. The patient was switched to dienogest therapy at age 35 and had hematuria and bloody stools at age 38. PET-CT revealed a 6-cm mass in the bladder with fluorodeoxyglucose accumulation and the diagnosis of endometriosis in the bladder, sigmoid colon, and cecum was confirmed after the biopsy result. The lesion’s tubular structures were positive for the estrogen receptor, but only 30% positive for the progesterone receptor, and the H1047R mutation in PIK3CA was found in tubular structures of the bladder lesion. GnRHa therapy caused the tumors to shrink.

**Conclusion:**

Decreased progesterone receptor expression and oncogenic mutations may influence the course of less common and rare site endometriosis. Rare site endometriosis often requires long-term hormone therapy, and management should be tailored to the patient's life stage, keeping in mind complications, such as decreased bone density.

## Background

Endometriosis occurs in 10% of sexually active women, 50% of infertile women, and 70% of women suffering from pelvic pain, and requires long-term management [1]. Endometriosis is exacerbated by estrogen and ameliorated by progesterone. Therefore, hormone therapy using gonadotropin releasing hormone agonist (GnRHa) or gonadotropin releasing hormone antagonist, or progesterone therapy using dienogest is recommended as standard therapy [2, 3]. Recent molecular analysis has revealed that deep endometriosis and ovarian endometriosis produce oncogenic mutations in many cases [4, 5]. Endometriosis rarely occurs in extragonadal organs, such as the bladder and intestinal tract, causing organ-specific symptoms. Knabben et al. reported that urinary tract endometriosis was present in 52.6% of patients suffering from deep infiltrating endometriosis [6]. In our literature search, no comprehensive reports on this condition were found except several case reports that included rather insufficient discussion on the optimal treatment and the related molecular analysis [7].

We report an extragonadal endometriosis case controlled by drug therapy for 14 years with analysis of the sex hormone receptor expression and PIK3CA mutation. The course and study of this case will aid in understanding this rare condition.

## Case presentation

The studied patient is currently 42-year-old, G0P0. Family history, Nothing noteworthy. Her menstrual period started at age of 13 years. Dysmenorrhea was diagnosed at age of 15 years. At age of 16 years, she had a fever, lower abdominal pain, and fullness of the abdomen. Computed tomography (CT) showed ovarian swelling, and laparotomy was performed. Endometriosis and an infected endometriotic cyst were identified in the abdominal cavity; therefore, ovarian cystectomy and lavage drainage were performed.

She visited our hospital at age of 29 years with increased menstrual pain. Low-dose estrogen/progestin (LEP) and non-steroidal anti-inflammatory drugs (NSAIDs) were administered. However, she experienced strong abdominal pains and underwent CT scan that revealed bilateral hydronephrosis. The blood test indicated white blood cells (WBC) 12.8 × 10^3^/uL (reference 3.3–8.6), C-reactive protein (CRP) 20.0 mg/dL (reference 0–0.1), blood urea nitrogen (BUN) 16 mg/dL (reference 8–20), creatinine (CRE) 1.02 mg/dL (reference 0.46–0.79) and urinary occult blood 1 + . The patient was manged by insertion of intravesical catheter and antibiotic. When the renal function improved and inflammation subsided the urinary catheter removed and the patient was discharged. Magnetic resonance imaging (MRI) performed after discharge revealed bladder wall thickening, and bladder endometriosis was diagnosed. At three months, CT scan showed that the hydronephrosis was worsened. A right ureteral stent was placed and the left ureteral stent placement was not possible. The patient showed no improvement in hydronephrosis even after stent implantation, and developed left abdominal pain. She had a fever of 39.0–39.9 °C with WBC 10.4 × 10^3^/uL, CRP 21.5 mg/dL, BUN 28 mg/dL and CRE 3.50 mg/dL. Acute pyelonephritis due to left ureteral obstruction and postrenal renal failure were suspected and a left nephrostomy was performed. However, right hydronephrosis and renal function did not improve, and one month after the left nephrostomy, the right nephrostomy was performed. Later, the renal fistula catheter was regularly replaced. Regarding endometriosis, the GnRHa therapy (leuprorelin acetate 1.88 mg/month) was continued with hormone add-back of conjugated estrogen 0.625 mg every other day until age of 35 years, when the therapy was changed to dienogest 2 mg/day. Her pelvic MRI at age of 36 years revealed only some irregularities of the bladder wall.

**Fig. 1:**
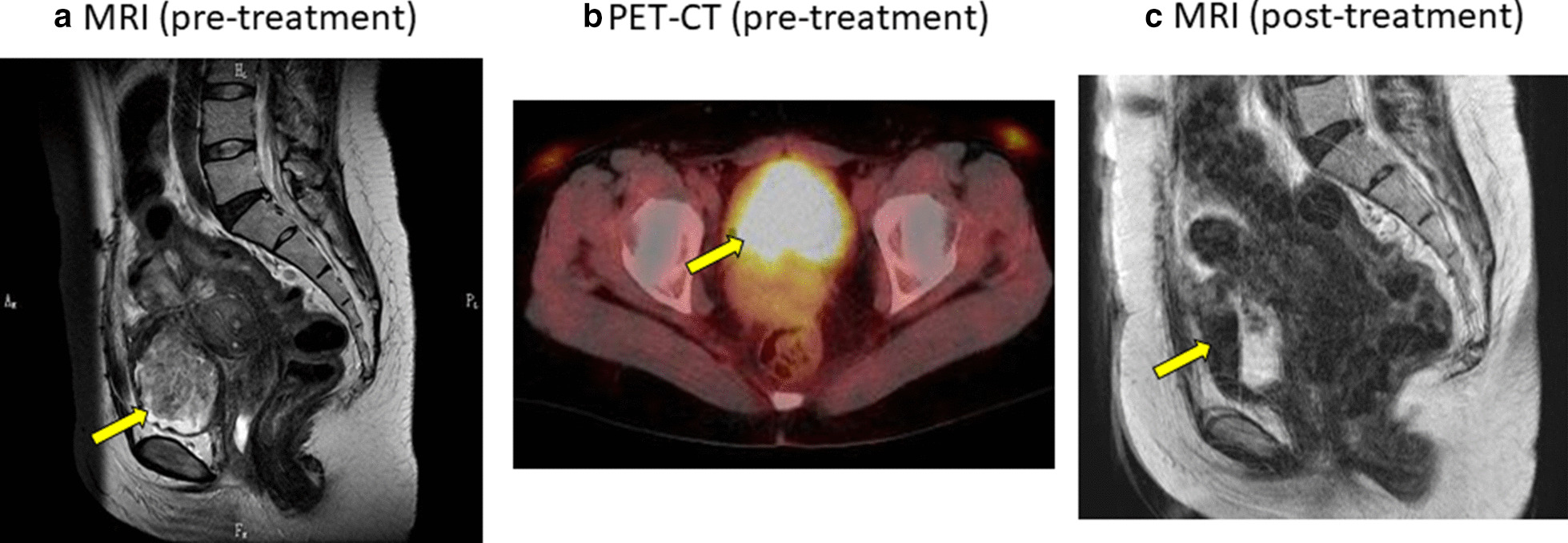
Bladder mass by **a** pre-treatment MRI (T2-weighted), **b** pre-treatment PET-CT, **c** post-treatment MRI (T2-weighted)

**Fig. 2:**
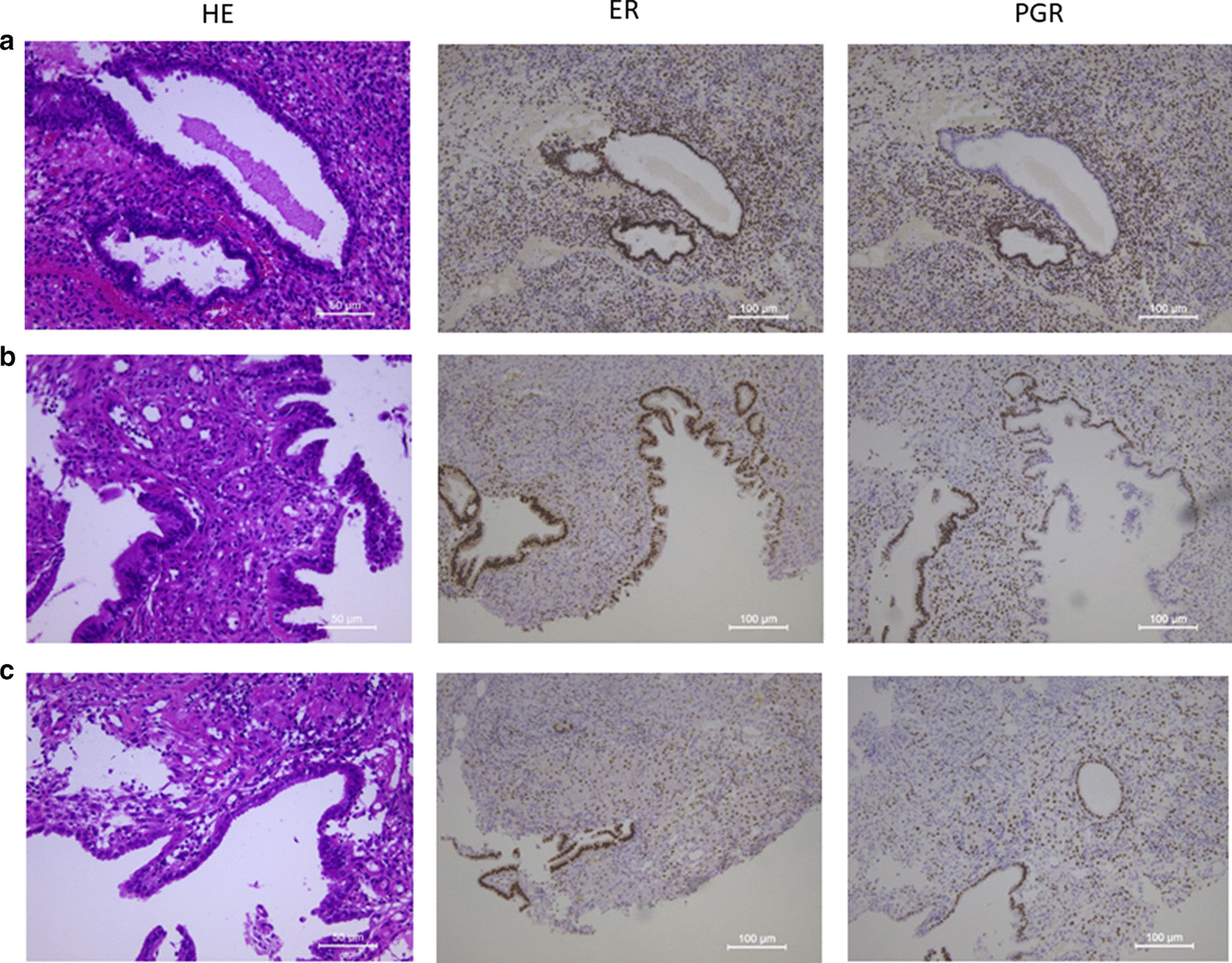
Pathology of the masses in **a** the bladder, **b** S-shaped colon, **c** cecus. Left row: Hematoxylin–Eosin Staining (× 200), middle row: estrogen receptor expression by immunohistochemistry (× 100), right row: progesterone receptor expression by immunohistochemistry (× 100)

**Fig. 3:**
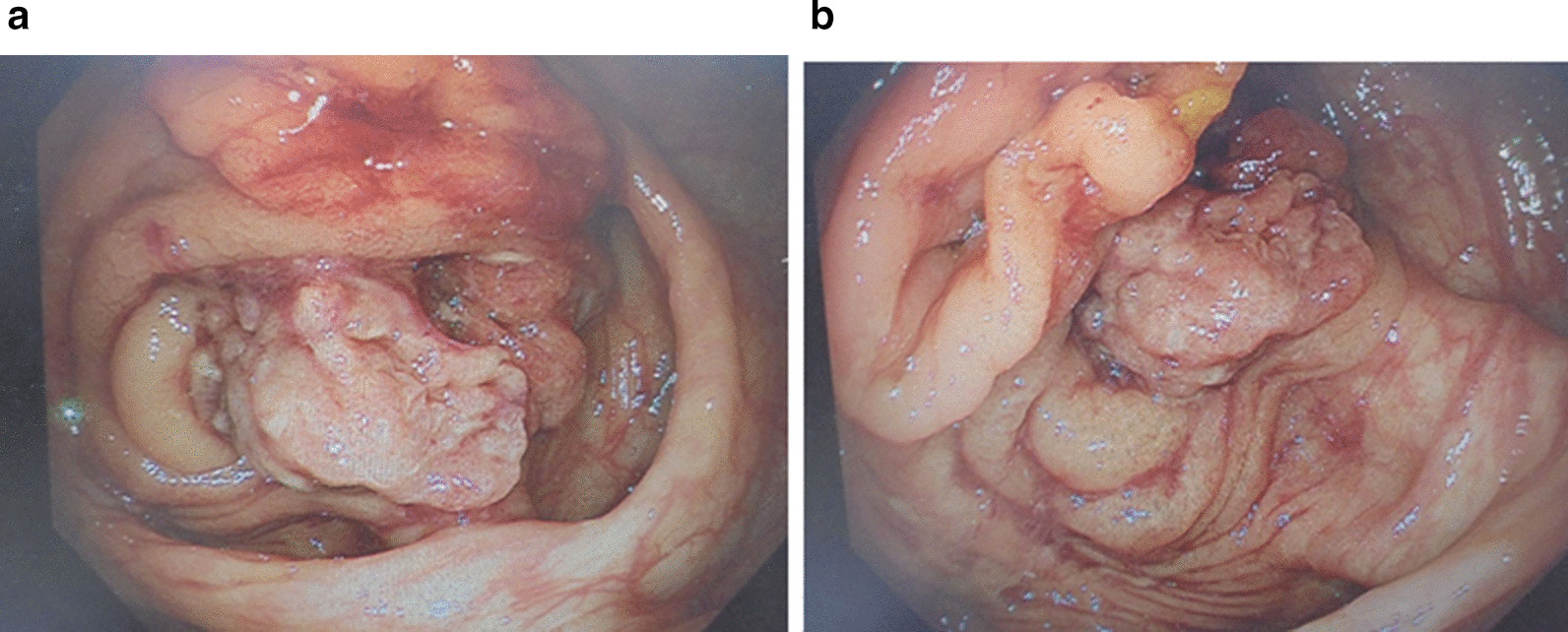
**a** Colonoscopy of the S-shaped colon mass, **b** colonoscopy of the cecal mass

At age of 38 years, she had hematuria and bloody stools, and MRI revealed 6 cm-sized mass in the bladder and 1.8 cm-sized mass in the cecum (Fig. 1a). Positron emission tomography and computed tomography (PET-CT) indicated standardized uptake value (SUV) max of 13.16 in the bladder tumor, and 3.43 in the cecum mass (Fig. 1b). Malignancy caused by endometriosis was suspected and a cystoscopic guided biopsy was performed that showed the tumor in the bladder was endometriosis (Fig. 2a). Colonoscopy revealed tumors in the cecum and sigmoid colon (Fig. 3a, b) which were demonstrated by biopsy to be endometriosis (Fig. 2b, c); endometriosis with tumor formation or polypoid endometriosis was diagnosed. Immunohistochemistry showed that the bladder and intestinal endometriotic lesions were estrogen receptor (ER) -positive in the tubular and interstitial parts (Fig. 2a–c). For progesterone receptor (PGR) expression, the lesions were positive in the interstitial parts, but only 30% in the tubular parts. Using FFPE specimens of lesions in the bladder, cecum, and sigmoid colon, the tubular parts were collected by laser microdissection according to the method described in the previous report [8], and three hot spots of PIK3CA (E542K, E545K, and H1047R) were examined by droplet digital PCR. The H1047R mutation was found only in the bladder lesion that was the largest.

As treatment, the surgical option was pelvic exenteration that was judged too invasive; therefore, the drug therapy was continued. Based on the history and immunostaining results, the lesion was judged progesterone-resistant but estrogen-dependent; therefore, GnRHa (leuprorelin acetate 1.88 mg) was administered once every 4 weeks for the first 6 months, resulting in a significant reduction of the intravesical mass volume confirmed by pelvic MRI (Fig. 1c). Later, GnRHa was administered once every 6 weeks, and the total number of administrations to date is 38 GnRHa injections. Bloody stool disappeared, and hematuria rarely appeared. Bone density is gradually decreasing, but no osteoporosis observed.

## Discussion

Bladder endometriosis, causing organ-specific symptoms, is observed in approximately 1% of all endometriosis cases, and the symptoms include dysuria and hematuria [9]. Knabben et al. reported that 68.8% of patients with bladder endometriosis have urinary symptoms [6]. In the present case, bilateral hydronephrosis was observed, probably due to the lower ureter stenosis caused by endometriosis [2]. In urinary tract endometriosis with hydronephrosis, ureteral stent placement is to be used only as adjunct during surgery. Therefore, ureteral stent placement did not improve hydronephrosis, and bilateral nephrostomy was eventually required in our case [2]. In surgery for bladder endometriosis, the lesion should be removed without residue. However, in our case, the bladder lesion is so widespread that surgical urinary tract reconstruction is considered difficult; therefore, bilateral renal fistula is maintained [2].

There have been multiple case reports showing that PET-CT reveals fluorodeoxyglucose (FDG) accumulation associated with endometriotic lesions, while there is a single report that found no prospective FDG accumulation in 10 endometriosis patients examined preoperatively [10–13]. In our case, a remarkable increase in intravesical mass was observed during the treatment course, resulting in so-called polypoid endometriosis, and significant accumulation of FDG was observed (Fig. 1b) [14]. To the best of our knowledge, this is the first report of PET-CT findings for polypoid endometriosis, which suggest that PET-CT may not be useful in differentiating tumor-forming polypoid endometriosis from malignant tumors.

In our case, the bladder lesion was exacerbated after switching to dienogest therapy. In the biopsy specimen at that time, PGR expression was observed only in approximately 30% of the endometriotic tubular structures (Fig. 2a–c), which might explain their resistance to the progesterone preparation. In endometriosis, progesterone resistance can be caused by molecular mechanisms, such as gene polymorphisms, altered microRNA expression, and epigenetic modifications of PGR [15]. In our case, tubular structures with and without PGR expression were found distinctively adjacent to each other, that may suggest the methylation of the PGR promoter [15]. On the other hand, ER expression found in 100% of the tubular structures may explain the successful GnRHa therapy. Thus, in endometriosis examining the expression of the sex hormone receptor by biopsy is considered useful for selecting the optimal hormone therapy.

Sequencing analysis of genes in deep endometriosis and endometriotic cyst revealed that oncogenic mutations frequently occur in endometriosis [3, 4]. Endometriotic lesions containing genetic mutations found at multiple distant sites were reported to be identical clones [3, 4]. Similarly, in our case, the endometriosis lesions in the bladder and intestinal tract are considered clones; however, the mechanism of this phenomenon comparable to cancer metastasis is unknown. To the best of our knowledge, this is the first report of gene mutation analysis in endometriosis observed in so-called extragonadal organs namely the bladder and intestine. The oncogenic mutation of PIK3CA was only detected in the bladder lesion, probably due to its lesion size and marked FDG uptake. It should be noted that oncogenic mutation frequently found in endometriosis does not immediately indicate susceptibility to carcinogenesis, and that the malignant transformation of bladder and ureteral endometriosis is extremely rare [2, 8, 9]. There is a review report that driver mutations in endometriosis are not necessarily synonymous with malignancy or precancerous lesions, and in light of this, the PIK3CA mutation observed in this case may be a passenger mutation rather than a driver mutation [16].

GnRHa therapy for more than 6 months accompanies bone density decrease. Our patient has been on GnRHa therapy for 11 years, except for 3 years on dienogest. The add-back therapy with estrogen performed during the first 6 years and every 6 weeks during the recent 5 years may help reduce the side effects of GnRHa. Currently, her bone density level is in the normal range, but close continued monitoring required.

## Conclusion

The present case report suggests that when endometriosis in the bladder or intestine diagnosed; (i) FDG accumulation on PET-CT should be investigated for visual differentiation from malignancy, (ii) ER and PGR expression on biopsy sample is useful to determine the course of treatment, and (iii) oncogenic mutations, such as PIK3CA mutation affect proliferation.

Rare site endometriosis often requires long-term hormone therapy, and management should be tailored to the patient's life stage, keeping in mind complications, such as decreased bone density.

## Data Availability

The datasets used and/or analysed during the current study available from the corresponding author on reasonable request.

## Abbreviations

GnRHa: Gonadotropin releasing hormone agonist
CT: Computed tomography
LEP: Low-dose estrogen/progestin
NSAIDs: Non-steroidal anti-inflammatory drugs
WBC: White blood cells
CRP: C-reactive protein
BUN: Blood urea nitrogen
CRE: Creatinine
MRI: Magnetic resonance imaging
PET-CT: Positron emission tomography and computed tomography
SUV: Standardized uptake value
ER: Estrogen receptor
PGR: Progesterone receptor
FDG: Fluorodeoxyglucose
